# Fibromyalgia and Depression

**DOI:** 10.1155/2012/486590

**Published:** 2011-11-19

**Authors:** Richard H. Gracely, Marta Ceko, M. Catherine Bushnell

**Affiliations:** ^1^Center for Neurosensory Disorders, University of North Carolina, CBNo.7280, 3330 Thurston Building, Chapel Hill, NC 27599, USA; ^2^Alan Edwards Centre for Research on Pain, McGill University, Montreal, QC, Canada; ^3^Integrated Program in Neuroscience, McGill University, Montreal, QC, Canada; ^4^Department of Anesthesia, McGill University, Montreal, QC, Canada; ^5^Department of Neurology & Neurosurgery, McGill University, Montreal, QC, Canada

## Abstract

Fibromyalgia and depression might represent two manifestations of affective spectrum disorder. They share similar pathophysiology and are largely targeted by the same drugs with dual action on serotoninergic and noradrenergic systems. Here, we review evidence for genetic and environmental factors that predispose, precipitate, and perpetuate fibromyalgia and depression and include laboratory findings on the role of depression in fibromyalgia. Further, we comment on several aspects of fibromyalgia which support the development of reactive depression, substantially more so than in other chronic pain syndromes. However, while sharing many features with depression, fibromyalgia is associated with somatic comorbidities and absolutely defined by fluctuating spontaneous widespread pain. Fibromyalgia may, therefore, be more appropriately grouped together with other functional pain disorders, while psychologically distressed subgroups grouped additionally or solely with affective spectrum disorders.

## 1. Introduction

The primary and debilitating symptom of fibromyalgia (FM) is widespread spontaneous pain. The ACR 1990 criteria also included a demonstration of tenderness. Recently revised diagnostic criteria have delegated the symptom of tenderness to a list of frequent symptoms that include fatigue, lack of refreshing sleep, cognitive impairments, abdominal discomfort, and headache [1]. This list also includes depression, which is prominent in fibromyalgia with a lifetime prevalence of about 90% for depressive symptoms and 62–86% for major depressive disorder (MDD) [2–5]. At any point in time, the best estimate of cooccurrence of depressive symptoms in FM is 40% [6]. The high occurrence of depression in FM has led to consideration of common pathophysiologic mechanisms and to the possible classification of fibromyalgia as one of the family of affective spectrum disorders that include many psychiatric conditions such as MDD, generalized anxiety disorder and posttraumatic stress disorder, and somatic conditions such as irritable bowel syndrome and migraine [3, 7, 8]. 

There is compelling evidence to link fibromyalgia and depression. They cooccur, they share similar pathophysiology, and the pharmacological treatment of each includes (but is not limited to) the same dual serotoninergic and noradrenergic agonists such as amitriptyline, duloxetine, and milnacipran. These similarities support the concept that depression and FM are “differential symptom presentations of a single underlying condition” [9–11]. 

## 2. Pathophysiology of Fibromyalgia and Depression: Predisposing, Precipitating, and Perpetuating Factors

The underlying processes of both depression and FM can be characterized by the lifetime course in an individual person. These processes can be organized by the three “P”s of predisposing, precipitating, and perpetuating factors. Considerable evidence suggests that genetic and environmental factors *predispose* individuals to develop depression or FM. Indeed, a fundamental property of the multiple genetic associations with depression is not that these genes cause depression but rather that they increase the risk of developing depression in response to a precipitating event [9]. The considerable evidence for increased vulnerability to depression includes genes involved in the function of serotonin, catecholamines, monoamines, CRF, glutamate, and brain-derived neurotrophic factor [9, 12–16]. The evidence suggests that these genes result in an intermediate phenotype that increases the general risk of a psychiatric disorder precipitated by an environmental stressor or other triggering event [9, 13]. A similar concept has been proposed for FM in which both genetic factors and environmental events predispose individuals to develop FM in response to a subsequent precipitating event. Genetic factors in FM are implicated by familial prevalence [6, 8, 17, 18]. Converging evidence suggests that a polymorphism in the serotonin transporter (5-HTT) gene, implicated in MDD, may also be implicated in FM [19, 20]. This genetic influence, the established influence of environment, and gene/environmental interactions may all predispose individuals to develop FM and depression. Many of the precipitating events described below, such as physical trauma or sexual abuse, also likely contribute to a predisposed state. 

Raphael et al. [18] have provided elegant evidence for the separate and joint predisposition to develop FM and MDD. In a community-based sample, they recruited individuals with both, either, or no MDD and FM, essentially filling 4 cells of a 2 × 2 table of FM presence (y/n) for one dimension and MDD presence (y/n) for the other dimension. These four cells defined subject categories, and the data of interest were collected from all available adult first-degree relatives of these subjects. Unlike previous studies that used reports of the primary subjects for data on relatives, this study actually interviewed the relatives. The results support a familial aggregation of FM and MDD. In comparison to a baseline rate of MDD of 28.7% in relatives of subjects without either MDD or FM, the rate of MDD in relatives was 39.0% in subjects with MDD and 37.3% in subjects with FM. Having both FM and MDD increased the rate of MDD in relatives to 45.5%. Expressed as odds ratios (ORs) in comparison to the groups that did not have either MDD or FM, the FM and MDD were similar with ORs of 1.47 and 1.56, and the combination of both FM and MDD increased the ORs of family MDD to 2.02. 

The results of Raphael et al. [18] were interpreted as support for FM as a depression spectrum disorder. Interestingly, the linkage for FM and MDD was not found for FM and any mood disorder excluding MDD. These results suggest a familial, likely genetic, linkage in the predisposition for acquiring FM and MDD and also suggest that depressive symptoms without MDD can be in reaction to the presence of FM and not due to a linked common mechanism.

Once predisposed to develop FM or depression, these syndromes can be *precipitated* by events ranging from injury to psychosocial stressors [21, 22]. Cited physical examples include physical trauma, illness, infections such as HIV, surgery, and autoimmune disease and motor vehicle accidents [7, 23]. Psychosocial stressors range from catastrophic events such as war to sexual abuse and other forms of emotional stress and trauma [7, 23]. Physical and psychosocial workplace events can also trigger these syndromes. Harkness et al. [24] documented precipitating physical workplace, events such as heavy lifting and repetitive motion, and psychosocial factors such as monotonous work and low social support.

Once triggered, both depression and FM involve a number of similar physiological mechanisms that likely perpetuate these disorders. It is well known that the acute response to stress, mobilizing the organism to deal with potentially life-threatening events, is beneficial if it remains acute. If abnormally prolonged, the disruption of normal bodily processes can lead to a number of disease states. The acute stress response, detailed in numerous reports, involves activation of the hypothalamic-pituitary-adrenal (HPA) axis which is coupled to the autonomic and limbic systems. Activation of the HPA system involves a chain of events. Corticotrophin-releasing hormone (CRH) is secreted by the hypothalamus, which results in pituitary secretion of ACTH. This CRH effect is synergistically augmented by hypothalamic secretion of argininevasopressin (AVP). Increased ACTH in turn stimulates adrenal secretion of cortisol. Cortisol is a potent glucocorticoid that activates cytoplasmic receptors throughout the body to ultimately mobilize action and inhibit vegetative processes such as reproduction and growth. The glucocorticoids also provide negative feedback regulation of the HPA axis via multiple pathways acting on the hypothalamus and pituitary. These effects of the HPA axis activation are integrated with the locus ceruleus-norepinephrine system (LCNE) that activates brain systems involved in affect and anticipation, precipitation, propagation and termination of stress-related activity and activation of pain [25].

The pathophysiology of both stress-induced depression and fibromyalgia has been described in terms of the deleterious consequences of an acute stress response that persists far beyond a normal duration, failing to “reset” after the stressor is removed or terminated. The results have been characterized by the effects on cortisol secretion, which interact with neurotransmitter systems and regulation of fatigue, affect, and pain, linking fibromyalgia and the subsets of depression described below. 

## 3. Reactive, Melancholic, and Atypical Depression

The term “depression” is used nonspecifically in the fibromyalgia literature similar to the nonspecific use of pain in the depression literature. The literature distinguishes between depressive symptoms, which can be evaluated by depression questionnaire instruments, and MDD, a diagnosis determined by an appropriately experienced clinician. Depression in fibromyalgia is not a unitary phenomenon. Depression can simply be a reaction to suffering from pain, compounded by the multiple comorbidities of fibromyalgia. In a broader sense, having any type of pain or just having a medical condition can lead to appropriate reactive depression. Depression may also take the form of a MDD that is physiologically linked to the mechanisms that perpetuate FM. In this case depression and FM might be considered to be parallel processes that share one or more predisposing, precipitating, and perpetuating features. These scenarios need not be mutually exclusive; depression and FM could be linked at numerous physiological and psychological levels that vary over the progression of the disorders. 

This complexity is compounded by heterogeneity in both FM and depression. Several studies have identified FM subgroups [26–31], finding major groups without psychological involvement and groups with considerable psychological distress. These subgroups respond differently to pharmacological treatments, for example, 5-HT3 receptor antagonists have been shown to be effective in patients without psychological distress and ineffective in patients with psychological distress [30]. The features of these subgroups and this differential response to treatment suggest that at least a subset of fibromyalgia patients may share features of an affective spectrum disorder.

Similar to fibromyalgia, diagnostic criteria distinguish three subtypes of MDD. The subtypes of melancholic and atypical depression characterize 60% of all MDD and are considered to be the prevalent types in FM [32, 33]. The third subtype is MDD with psychotic features. A recent study distinguished between melancholic and atypical depression in 76 fibromyalgia patients and observed that 40 met the criteria for atypical depression and 27 met the criteria for melancholic depression [32]. This distinction was not associated with differences in FM characteristics save for greater severity of depression in the melancholic depression group. 

## 4. Maturation of FM and Depression: From Hypercortisolism and Melancholic Depression to Hypocortisolism and Atypical Depression

The presence of melancholic and atypical depression in FM may represent a disease progression that also explains variable findings of HPA axis function in FM. These subtypes of MDD are associated with different alterations of HPA axis function; melancholic depression is associated with augmented cortisol (hypercortisolism) response while atypical depression is associated with a blunted secretion of cortisol (hypocortisolism). Gold et al. [33] have proposed a progression in fibromyalgia in which the early stage is associated with hypercortisolism incorporated with a prolonged normal stress response and the greater depression severity of melancholic depression. Over time, this augmented cortisol response is blunted to below normal levels, leading to hypcortisolism and the clinical features of atypical depression. This progression to ultimately atypical depression and the preponderance of women in both FM and atypical depression may lead to the finding of increased frequency of atypical depression in FM [32] and present implications for treatments targeted to the stages in this progression.

## 5. Scenarios of Linked FM and Depression

A physiological progression in FM and a transition from a melancholic to an atypical depression suggest one of several possible links between FM and depression. Aguglia et al. [2] consider three possibilities. The first is the reactive case in which the presence of a chronic painful and disabling disorder is a depressing event. The authors reject this hypothesis because the incidence of depression in FM [3, 34] is considerably higher than that in other comparable pain conditions or diseases. We offer counter arguments below.

Aguglia et al.'s [2] second possibility is that FM reflects a subthreshold depression, an idea that they reject since many FM patients are not depressed and do not become depressed. 

Aguglia et al.'s [2] third possibility is that FM and depression can be conceptualized as disorders with multiple peripheral and central manifestations that might belong to the same affective spectrum [8]. The proposed underlying mechanisms that perpetuate this spectrum include the mechanisms discussed above: alteration in HPA axis function, altered serotoninergic and noradrenergic function, and altered function of systems that involve substance P, neurosteroids, and cytokines.

## 6. Laboratory Evidence for the Role of Depression in FM

A study by Bartley et al. [35] explored the association of mood and fibromyalgia by examining the effect of FM on affective processing. Both FM patients and healthy controls, 17 per group, viewed pleasant, neutral, and unpleasant images and rated the pleasantness/unpleasantness and arousal evoked by the pictures. A number of physiological variables were recorded, and two-thirds of the pictures were accompanied by white noise stimuli to induce eye blinks. In comparison to controls, FM patients showed greater negative ratings, arousal, and EMG responses to the negative unpleasant images but no differences in responses to the pleasant images. These results, found also for olfactory stimuli [36], suggest that the mechanisms that modulate mood in FM do not result in an anhedonia, that is, a diminished ability to experience pleasure, but rather increase the unpleasantness of unpleasant material.

A number of laboratory studies have explored the interaction of depression and evoked pain sensitivity in fibromyalgia. Several have examined sensitivity to electrical stimuli at the spinal level. In an extension of previous findings of enhanced nociceptive flexion reflex (NFR) in fibromyalgia [37, 38], Ang et al. [39] explored the role of depression in the NFR in 32 women with FM. The Patient Health Questionnaire 8-item Depression Scale (PHQ-8) was used to divide subjects into depressed (score > 9) and nondepressed groups. An inverse association between the NFR threshold and ratings of current pain was observed in the nondepressed group (−3.9 mA change in NFR threshold per unit of current pain) but not in the depressed group (0.07 mA change in NFR threshold per unit of current pain). These results suggest that the presence of depression disrupted the positive relationship between ratings of current pain and NFR sensitivity in FM.

Neuroimaging studies have examined the influence of depression on supraspinal responses to painful pressure stimulation. Giesecke et al. [40] used the fMRI BOLD method to evaluate cerebral responses evoked by painful pressure applied to the thumb in 30 patients with FM. Depression was assessed by the Center for Epidemiological Studies Depression Scale (CES-D) and the Composite International Diagnostic Interview (CIDI). Two analyses were performed on the data. In the first correlational analysis, the brain activity evoked by a subjectively similar pressure stimulus was computed for each subject for each voxel of the brain. For each of the 30 subject scores at each voxel, a linear regression was performed between each patient's pain-evoked fMRI activity and each patient's depression score on the CES-D. This resulted in a scatter plot in which each point is a person, defined by their depression score and the pain-evoked activity at that brain location. Correcting appropriately for multiple comparisons, there were significant associations in bilateral amygdala and in contralateral insula. In the second, intra-group, analysis, 7 FM patients diagnosed with MDD were matched to 7 FM patients without MDD and to 7 healthy control subjects. Equally painful stimuli, evoked by significantly less pressure in the FM groups, revealed activations in the pain matrix observed in the full group analysis above and in previous studies of painful thumb pressure pain. In addition, in the MDD group, these stimuli evoked activations in bilateral amygdala and contralateral insula, regions implicated in affective processing. The results suggest that the presence of depression had no effect on the sensory-discriminative processing of pain stimulation and had a selective effect on brain regions that process the affective-motivational dimension of pain.

An additional study in this laboratory used a correlational analysis to assess the influence of catastrophizing, a putatively depression-driven cognitive style of attaching overly negative interpretations to events [41, 42]. Since some argue that this style is a variant of depression [43, 44], the analysis controlled for depression, identifying brain regions of evoked pain activity associated with a measure of catastrophizing (Coping Strategies Questionnaire). [43] Applying an analysis strategy described above for Giesecke et al. [40], the analysis both identified brain regions, in which activation by painful thumb pressure was associated with catastrophizing, and regions in which activation in response to painful pressure was significantly different in groups with low or high catastrophizing scores divided by a median split. The results identified several brain regions in which pain evoked by pressure to the thumb was associated with catastrophizing over a sample of 29 FM patients. These regions include contralateral anterior cingulate cortex, medial frontal gyrus, lentiform and ipsilateral middle frontal gyrus, secondary somatosensory cortex, claustrum, and cerebellum. These regions did not overlap with the brain regions (amygdala and insula) associated with depression in the study by Giesecke et al. [40], likely because the analysis controlled for the effect of depression [41]. Significantly greater activations in high versus low catastrophizing groups were observed in contralateral medial frontal gyrus and inferior parietal lobule, and in ipsilateral medial frontal gyrus, superior frontal gyrus, secondary somatosensory cortex, and anterior cingulate cortex. One region, ipsilateral inferior parietal lobule, showed significantly greater activation in the low catastrophizing group. Both analyses found effects in contralateral medial frontal gyrus and in ipsilateral secondary somatosensory cortex. These combined results suggest that catastrophizing is uniquely associated with brain regions involved in attention to pain and pain affective processing.

In another fMRI study in FM patients, depression and catastrophizing were not associated with the effects of pressure stimulation in any brain region. [45]. However, this study used randomly delivered stimuli of much shorter duration, which might have served to maximize the sensory saliency, and minimize the unpleasantness, of the stimulus-evoked sensations.

## 7. Treatment Evidence from Clinical Trials

Depression and FM also appear to be linked because drugs with dual serotoninergic and noradrenergic action are used to treat both conditions. The two prime examples are the noradrenergic, serotoninergic reuptake inhibitors (NSRIs) duloxetine and milnacipran, antidepressants approved recently in the US for treatment of FM. These agents mimic the dual action of amitriptyline, a tricyclic antidepressant which has long been used to treat chronic pain, including FM. One significant advantage is that these recent agents have a superior adverse-effect profile since they do not possess the well-known anticholinergic effects of amitriptyline. 

Classic analgesic studies of amitriptyline suggest that the pain-relieving mechanism is independent of the antidepressant effects. Pain relief is accomplished at a much smaller dose (25 mg) than those used for depression (100–150 mg), and analgesic effects are observed after one week of treatment while antidepressant effects were usually observed after three weeks of treatment. In addition, the pain-relieving effects are independent of the effects on depression and are observed in both depressed and nondepressed patients [46, 47]. The recent trials of duloxetine and milnacipran also provide evidence for a pain-relieving action of dual serotoninergic and noradrenergic agonists, that is, independent of depression [48–51]. A recent secondary analysis of 4 clinical trials of duloxetine in fibromyalgia explored treatment effects in patients who were comorbid with MDD. This analysis demonstrated the relative independence of MDD and fibromyalgia in that the baseline level of one did not affect the treatment efficacy of the other [4]. A path analysis also found predominant direct effects of treatment on mood and pain. These results suggest that the effects of the NSRI duloxetine on pain and MDD were mediated by independent mechanisms. However, the path analyses also showed indirect effects in which improved mood resulted in improved pain and improved pain resulted in improved mood. In the case of pain with unpleasantness and affective dimensions, the indirect effects of mood are reasonable and practically expected. Similarly, relieved pain can be expected to improve mood.

These indirect effects may reflect the influence of reactive depression elements in FM. Aguglia et al. [2] reject this scenario above because FM has a higher incidence of depression than similarly severe pain conditions or diseases. However, we feel that this issue deserves close scrutiny. Although comparative diseases (such as rheumatoid arthritis) may be comparable on severity, they may not be comparable on other disease features that more powerfully drive reactive depression. There are several aspects of FM that would fuel an augmented reactive depression in comparison to many other pain or medical syndromes. The pain is widespread, including by definition all four body quadrants and the axial skeleton. Pain extent is likely associated with the incidence and severity of depression. Manchikanti et al. [52] have shown that the incidence of MDD was 4% in a control group, 20% in a group with chronic pain in region of the body, and 32% in patients experiencing pain in more than one body region. This type effect might be assumed to be the highest in FM, in which the whole body pain encompassing 5 major regions would place it high on the function relating MDD to a number of body sites.

In addition to the extent, FM is characterized by significant spontaneous pain. In contrast, many pain conditions are characterized less by spontaneous pain and more by movement-evoked pain. The inability to alleviate pain by quiescence or postural adjustment may contribute to depression. Similarly, the extensive spontaneous pain of FM is accompanied by a large number of symptoms and comorbidities that likely contribute to the probability of reactive depression.

Furthermore, unlike other conditions such as rheumatoid arthritis or pain conditions with physical correlates, patients with fibromyalgia suffer symptoms with minimal objective signs. Many in the medical field still deny the very existence of FM, relegating it to general somatization or other psychiatric processes. Patients feel that they are not believed and must fight for acceptance. This lack of credible objective evidence for diagnosis or underlying mechanism likely explain the enthusiastic embrace of recent findings such as altered concentrations of substance P in CSF [53–56] and evidence of altered CNS processing at spinal [37, 38] and supraspinal [37, 38, 40, 48, 53–77] levels. It also is a very reasonable explanation for increased frequency and severity of reactive depression in FM.

Finally, in concert with the lack of objective signs, patients with fibromyalgia live with increased uncertainty of what they have and their prospects for the future. Since many features of depression are future-oriented (despair, hopelessness), this uncertainty is likely linked to depression.

## 8. Summary

In summary, depression and FM share both common features and differences at multiple physiological and psychological levels. Clear evidence indicates a mutual predisposition; for example, biologically based MDD is predicted nearly equally by the presence of familial MDD or FM. This mutual predisposition is likely due to a combination of genetic and environmental factors [12, 19, 78–82]. These predisposed individuals are particularly vulnerable to a precipitating event that triggers FM or MDD. An injury, motor vehicle accident, illness, or psychosocial stressor can activate a cascade of events with potent, persistent consequences. There is considerable support for the hypothesis that these precipitating events trigger an appropriate acute stress response, including increased CRH, AVP, ACTH, and cortisol. The initial elevations in cortisol are associated with a melancholic depression, and a gradual shift to hypocortisol levels coincides with a shift from melancholic depressive disorder to atypical depression disorder. FM has been characterized by both lowered and elevated wakening cortisol levels and associated with both melancholic and atypical depression [81, 83, 84]. The HPA axis system is complex with multiple regulating feedback loops. Although predisposed by similar mechanisms, FM and MDD may be mediated by distinctly different alterations of HPA function. For example, depressed mood may be due to internal dysregulation of the HPA system while the persistent pain of FM may be due to known cytokine-mediated HPA activation [85–87]. Similarly, involvement of systems mediated by serotonin and norepinephrine may be common to both FM and depression, yet the differential effects of NSRI and tricyclic treatments suggest at least distinct differences in the functioning of these systems in these two disorders.

FM and depression are clearly intertwined at the experiential and measurement level. An examination of depression questionnaires reveals numerous somatic items that would be endorsed by a person suffering from chronic pain. Depression hurts or at least provokes responses similar to hurt; for example, “I do not feel as well as I used to,” “I ache all over.” Conversely, to have pain is depressing, and the behavioral consequences of reduced activity and social isolation compound negative mood. FM is not fully endorsed by all clinicians and is mediated by unknown mechanisms with unknown prognosis. The patient's current state is unknown and uncertain, and the future more so. A persistent, intractable pain syndrome fuels helplessness and despair. The interactions can become cyclical and self-perpetuating. When treatments such as exercise or cognitive behavioral methods are shown to be effective, depression can rob the initiative and the ability to comply with such treatments.

FM has been included in the family of affective spectrum disorders (ASDs) that include other physical conditions such as migraine and irritable bowel syndrome and numerous psychiatric disorders such as attention-deficit/hyperactivity disorder, bulimia nervosa, dysthymic disorder, generalized anxiety disorder, major depressive disorder (MDD), obsessive compulsive disorder, panic disorder, posttraumatic stress disorder, premenstrual dysphoric disorder, and social phobia [3, 7, 8]. While sharing features such as predisposition to develop psychiatric conditions such as MDD, FM also is associated with a number of somatic comorbid conditions. With the predominant symptom of widespread pain, FM may be grouped also with other functional pain disorders such as temporomandibular pain disorders, Gulf War syndrome, and vulvodynia as well as irritable bowel syndrome and migraine. All of these may be expected to have some effects on common physiological systems such as dysregulation of the HPA axis, autonomic functions, and of systems regulating serotonin and norepinephrine. This commonality may reflect both the widespread role of these systems in health and disease and the limited repertoire of physiological responses to multiple sources of pathophysiology. Disparate mechanisms may result in similar symptoms, representing components of a stereotypic stress response as described by Selye [88]. The independence of treatment effects suggests that depression and FM are mediated by largely independent mechanisms with mutual modulation of specific symptoms. The widespread pain that characterizes FM suggests association with a family of somatic disorders, while psychologically distressed subgroups, requiring different treatment, may represent the additional or sole association with a family of affective spectrum disorders.

## References

[1] Wolfe F, Clauw DJ, Fitzcharles M-A (2011). Fibromyalgia criteria and severity scales for clinical and epidemiological studies: a modification of the ACR preliminary diagnostic criteria for fibromyalgia. *Journal of Rheumatology*.

[2] Aguglia A, Salvi V, Maina G, Rossetto I, Aguglia E (2010). Fibromyalgia syndrome and depressive symptoms: comorbidity and clinical correlates. *Journal of Affective Disorders*.

[3] Arnold LM, Hudson JI, Keck PE, Auchenbach MB, Javaras KN, Hess EV (2006). Comorbidity of fibromyalgia and psychiatric disorders. *Journal of Clinical Psychiatry*.

[4] Marangell LB, Clauw DJ, Choy E (2011). Comparative pain and mood effects in patients with comorbid fibromyalgia and major depressive disorder: secondary analyses of four pooled randomized controlled trials of duloxetine. *Pain*.

[5] Wilke WS, Gota CE, Muzina DJ (2010). Fibromyalgia and bipolar disorder: a potential problem?. *Bipolar Disorders*.

[6] Kato K, Sullivan PF, Evengård B, Pedersen NL (2006). Importance of genetic influences on chronic widespread pain. *Arthritis and Rheumatism*.

[7] Bradley LA (2009). Pathophysiology of fibromyalgia. *American Journal of Medicine*.

[8] Hudson JI, Arnold LM, Keck PE, Auchenbach MB, Pope HG (2004). Family study of fibromyalgia and affective spectrum disorder. *Biological Psychiatry*.

[9] Maletic V, Raison CL (2009). Neurobiology of depression, fibromyalgia and neuropathic pain. *Frontiers in Bioscience*.

[10] Cannon DM, Klaver JM, Peck SA, Rallis-Voak D, Erickson K, Drevets WC (2009). Dopamine type-1 receptor binding in major depressive disorder assessed using positron emission tomography
and [^11^C]NNC-112. *Neuropsychopharmacology*.

[11] Kendler KS, Gardner CO, Gatz M, Pedersen NL (2007). The sources of co-morbidity between major depression and generalized anxiety disorder in a Swedish national twin sample. *Psychological Medicine*.

[12] Bradley RG, Binder EB, Epstein MP (2008). Influence of child abuse on adult depression: moderation by the corticotropin-releasing hormone receptor gene. *Archives of General Psychiatry*.

[13] Dreher JC, Kohn P, Kolachana B, Weinberger DR, Berman KF (2009). Variation in dopamine genes influences responsivity of the human reward system. *Proceedings of the National Academy of Sciences of the United States of America*.

[14] Pezawas L, Meyer-Lindenberg A, Goldman AL (2008). Evidence of biologic epistasis between BDNF and SLC6A4 and implications for depression. *Molecular Psychiatry*.

[15] Caspi A, Sugden K, Moffitt TE (2003). Influence of life stress on depression: moderation by a polymorphism in the 5-HTT gene. *Science*.

[16] Kendler KS, Kuhn JW, Vittum J, Prescott CA, Riley B (2005). The interaction of stressful life events and a serotonin transporter polymorphism in the prediction of episodes of major depression: a replication. *Archives of General Psychiatry*.

[17] Arnold LM, Hudson JI, Janal MN, Nayak S, Schwartz JE, Gallagher RM (2004). Comment on “Familial aggregation of depression in fibromyalgia: a community-based test of alternative hypotheses, Raphael et al., Pain 110 (2004) 449–460”. *Pain*.

[18] Raphael KG, Janal MN, Nayak S, Schwartz JE, Gallagher RM (2004). Familial aggregation of depression in fibromyalgia: a community-based test of alternate hypotheses. *Pain*.

[19] Cohen H, Buskila D, Neumann L, Ebstein RP (2002). Confirmation of an association between fibromyalgia and serotonin transporter promoter region (5-HTTLPR) polymorphism, and relationship to anxiety-related personality traits. *Arthritis and Rheumatism*.

[20] Offenbaecher M, Bondy B, de Jonge S (1999). Possible association of fibromyalgia with a polymorphism in the serotonin transporter gene regulatory region. *Arthritis and Rheumatism*.

[21] Blackburn-Munro G, Blackburn-Munro RE (2001). Chronic pain, chronic stress and depression: coincidence or consequence?. *Journal of Neuroendocrinology*.

[22] Johnson L, Andersson-Lundman G, Åberg-Wistedt A, Mathé AA (2000). Age of onset in affective disorder: its correlation with hereditary and psychosocial factors. *Journal of Affective Disorders*.

[23] Mease P (2005). Fibromyalgia syndrome: review of clinical presentation, pathogenesis, outcome measures, and treatment. *Journal of Rheumatology*.

[24] Harkness EF, Macfarlane GJ, Nahit E, Silman AJ, McBeth J (2004). Mechanical injury and psychosocial factors in the work place predict the onset of widespread body pain: a two-year prospective study among cohorts of newly employed workers. *Arthritis and Rheumatism*.

[25] Tsigos C, Chrousos GP (2002). Hypothalamic-pituitary-adrenal axis, neuroendocrine factors and stress. *Journal of Psychosomatic Research*.

[26] de Souza JB, Goffaux P, Julien N, Potvin S, Charest J, Marchand S (2009). Fibromyalgia subgroups: profiling distinct subgroups using the Fibromyalgia Impact Questionnaire. A preliminary study. *Rheumatology International*.

[27] Giesecke T, Williams DA, Harris RE (2003). Subgrouping of fibromyalgia patients on the basis of pressure-pain thresholds and psychological factors. *Arthritis and Rheumatism*.

[28] Müller W, Schneider M, Joos T, Hsu HY, Stratz T (2007). Subgroups of fibromyalgia. *Schmerz*.

[29] Calandre EP, Garcia-Carrillo J, Garcia-Leiva JM, Rico-Villademoros F, Molina-Barea R, Rodriguez-Lopez CM Subgrouping patients with fibromyalgia according to the results of the fibromyalgia impact questionnaire: a replication study.

[30] Seidel MF, Müller W (2011). Differential pharmacotherapy for subgroups of fibromyalgia patients with specific consideration of 5-HT3 receptor antagonists. *Expert Opinion on Pharmacotherapy*.

[31] Thieme K, Turk DC, Flor H (2004). Comorbid Depression and anxiety in fibromyalgia syndrome: relationship to somatic and psychosocial variables. *Psychosomatic Medicine*.

[32] Ross RL, Jones KD, Ward RL, Wood LJ, Bennett RM (2010). Atypical depression is more common than melancholic in fibromyalgia: an observational cohort study. *BMC Musculoskeletal Disorders*.

[33] Gold PW, Gabry KE, Yasuda MR, Chrousos GP (2002). Divergent endocrine abnormalities in melancholic and atypical depression: clinical and pathophysiologic implications. *Endocrinology and Metabolism Clinics of North America*.

[34] Gormsen L, Rosenberg R, Bach FW, Jensen TS (2010). Depression, anxiety, health-related quality of life and pain in patients with chronic fibromyalgia and neuropathic pain. *European Journal of Pain*.

[35] Bartley EJ, Rhudy JL, Williams AE (2009). Experimental assessment of affective processing in fibromyalgia. *Journal of Pain*.

[36] Schweinhardt P, Sauro KM, Bushnell MC (2008). Fibromyalgia: a disorder of the brain?. *Neuroscientist*.

[37] Banic B, Petersen-Felix S, Andersen OK (2004). Evidence for spinal cord hypersensitivity in chronic pain after whiplash injury and in fibromyalgia. *Pain*.

[38] Desmeules JA, Cedraschi C, Rapiti E (2003). Neurophysiologic evidence for a central sensitization in patients with fibromyalgia. *Arthritis and Rheumatism*.

[39] Ang DC, Chakr R, France CR (2011). Association of nociceptive responsivity with clinical pain and the moderating effect of depression. *Journal of Pain*.

[40] Giesecke T, Gracely RH, Williams DA, Geisser ME, Petzke FW, Clauw DJ (2005). The relationship between depression, clinical pain, and experimental pain in a chronic pain cohort. *Arthritis and Rheumatism*.

[41] Gracely RH, Geisser ME, Giesecke T (2004). Pain catastrophizing and neural responses to pain among persons with fibromyalgia. *Brain*.

[42] Edwards RR, Calahan C, Mensing G, Smith M, Haythornthwaite JA (2011). Pain, catastrophizing, and depression in the rheumatic diseases. *Nature Reviews Rheumatology*.

[43] Rosenstiel AK, Keefe FJ (1983). The use of coping strategies in chronic low back pain patients: relationship to patient characteristics and current adjustment. *Pain*.

[44] Sullivan MJL, D’Eon JL (1990). Relation between catastrophizing and depression in chronic pain patients. *Journal of Abnormal Psychology*.

[45] Jensen KB, Petzke F, Carville S (2010). Anxiety and depressive symptoms in fibromyalgia are related to poor perception of health but not to pain sensitivity or cerebral processing of pain. *Arthritis and Rheumatism*.

[46] Max MB, Culnane M, Schafer SC (1987). Amitriptyline relieves diabetic neuropathy pain in patients with normal or depressed mood. *Neurology*.

[47] Max MB, Lynch SA, Muir J, Shoaf SE, Smoller B, Dubner R (1992). Effects of desipramine, amitriptyline, and fluoxetine on pain in diabetic neuropathy. *The New England Journal of Medicine*.

[48] (1990). 1990 classification criteria of fibromyalgia from the American College of Rheumatology. Report of the Multicenter Criteria Committee. *Union Medicale du Canada*.

[49] Arnold LM, Lu Y, Crofford LJ (2004). A double-blind, multicenter trial comparing duloxetine with placebo in the treatment of fibromyalgia patients with or without major depressive disorder. *Arthritis and Rheumatism*.

[50] Arnold LM, Rosen A, Pritchett YL (2005). A randomized, double-blind, placebo-controlled trial of duloxetine in the treatment of women with fibromyalgia with or without major depressive disorder. *Pain*.

[51] Gendreau RM, Thorn MD, Gendreau JF (2005). Efficacy of milnacipran in patients with fibromyalgia. *Journal of Rheumatology*.

[52] Manchikanti L, Pampati V, Beyer C, Damron K (2002). Do number of pain conditions influence emotional status?. *Pain Physician*.

[53] Reynolds WJ, Chiu B, Inman RD (1988). Plasma substance P levels in fibrositis. *Journal of Rheumatology*.

[54] Russell IJ, Orr MD, Littman B (1994). Elevated cerebrospinal fluid levels of substance P in patients with the fibromyalgia syndrome. *Arthritis and Rheumatism*.

[55] Schwarz MJ, Späth M, Müller-Bardorff H, Pongratz DE, Bondy B, Ackenheil M (1999). Relationship of substance P, 5-hydroxyindole acetic acid and tryptophan in serum of fibromyalgia patients. *Neuroscience Letters*.

[56] Vaeroy H, Helle R, Forre O, Kass E, Terenius L (1988). Elevated CSF levels of substance P and high incidence of Raynaud phenomenon in patients with fibromalgia: new features for diagnosis. *Pain*.

[57] Cook DB, Lange G, Ciccone DS, Liu WC, Steffener J, Natelson BH (2004). Functional imaging of pain in patients with primary fibromyalgia. *Journal of Rheumatology*.

[58] Gracely RH, Petzke F, Wolf JM, Clauw DJ (2002). Functional magnetic resonance imaging evidence of augmented pain processing in fibromyalgia. *Arthritis and Rheumatism*.

[59] Burgmer M, Pogatzki-Zahn E, Gaubitz M (2010). Fibromyalgia unique temporal brain activation during experimental pain: a controlled fMRI Study. *Journal of Neural Transmission*.

[60] Burgmer M, Pogatzki-Zahn E, Gaubitz M, Wessoleck E, Heuft G, Pfleiderer B (2009). Altered brain activity during pain processing in fibromyalgia. *NeuroImage*.

[61] Diers M, Koeppe C, Yilmaz P (2008). Pain ratings and somatosensory evoked responses to repetitive intramuscular and intracutaneous stimulation in fibromyalgia syndrome. *Journal of Clinical Neurophysiology*.

[62] Gibson SJ, Littlejohn GO, Gorman MM, Helme RD, Granges G (1994). Altered heat pain thresholds and cerebral event-related potentials following painful CO2 laser stimulation in subjects with fibromyalgia syndrome. *Pain*.

[63] Gunduz B, Bayazit YA, Celenk F (2008). Absence of contralateral suppression of transiently evoked otoacoustic emissions in fibromyalgia syndrome. *Journal of Laryngology and Otology*.

[64] Hargrove JB, Bennett RM, Simons DG, Smith SJ, Nagpal S, Deering DE (2010). Quantitative electroencephalographic abnormalities in fibromyalgia patients. *Clinical EEG and Neuroscience*.

[65] Harris RE, Clauw DJ, Scott DJ, McLean SA, Gracely RH, Zubieta JK (2007). Decreased central *μ*-opioid receptor availability in fibromyalgia. *Journal of Neuroscience*.

[66] Harris RE, Sundgren PC, Craig AD (2009). Elevated insular glutamate in fibromyalgia is associated with experimental pain. *Arthritis and Rheumatism*.

[67] Jensen KB, Kosek E, Petzke F (2009). Evidence of dysfunctional pain inhibition in Fibromyalgia reflected in rACC during provoked pain. *Pain*.

[68] Lorenz J (1998). Hyperalgesia or hypervigilance? An evoked potential approach to the study of fibromyalgia syndrome. *Zeitschrift für Rheumatologie*.

[69] Lutz J, Jäger L, de Quervain D (2008). White and gray matter abnormalities in the brain of patients with fibromyalgia: a diffusion-tensor and volumetric imaging study. *Arthritis and Rheumatism*.

[70] Mhalla A, de Andrade DC, Baudic S, Perrot S, Bouhassira D (2010). Alteration of cortical excitability in patients with fibromyalgia. *Pain*.

[71] Montoya P, Sitges C, García-Herrera M (2006). Reduced brain habituation to somatosensory stimulation in patients with fibromyalgia. *Arthritis and Rheumatism*.

[72] Pujol J, López-Solà M, Ortiz H (2009). Mapping brain response to pain in fibromyalgia patients using temporal analysis of fMRI. *PLoS One*.

[73] Schmidt-Wilcke T, Luerding R, Weigand T (2007). Striatal grey matter increase in patients suffering from fibromyalgia—a voxel-based morphometry study. *Pain*.

[74] Sundgren PC, Petrou M, Harris RE (2007). Diffusion-weighted and diffusion tensor imaging in fibromyalgia patients: a prospective study of whole brain diffusivity, apparent diffusion coefficient, and fraction anisotropy in different regions of the brain and correlation with symptom severity. *Academic Radiology*.

[75] Wood PB, Glabus MF, Simpson R, Patterson JC (2009). Changes in gray matter density in fibromyalgia: correlation with dopamine metabolism. *Journal of Pain*.

[76] Yunus MB, Young CS, Saeed SA, Mountz JM, Aldag JC (2004). Positron emission tomography in patients with fibromyalgia syndrome and healthy controls. *Arthritis Care and Research*.

[77] Vaeroy H, Sakurada T, Forre O, Kass E, Terenius L (1989). Modulation of pain in fibromyalgia (fibrositis syndrome) cerebrospinal fluid (CSF) investigation of pain related neuropeptides with special reference to calcitonin gene related peptide (CGRP). *Journal of Rheumatology*.

[78] Cohen H, Neumann L, Glazer Y, Ebstein RP, Buskila D (2009). The relationship between a common catechol-O-methyltransferase (COMT) polymorphism val158met and fibromyalgia. *Clinical and Experimental Rheumatology*.

[79] Jabbi M, Kema IP, Van Der Pompe G, Te Meerman GJ, Ormel J, Den Boer JA (2007). Catechol-o-methyltransferase polymorphism and susceptibility to major depressive disorder modulates psychological stress response. *Psychiatric Genetics*.

[80] McLean SA, Williams DA, Stein PK (2006). Cerebrospinal fluid corticotropin-releasing factor concentration is associated with pain but not fatigue symptoms in patients with fibromyalgia. *Neuropsychopharmacology*.

[81] Weissbecker I, Floyd A, Dedert E, Salmon P, Sephton S (2006). Childhood trauma and diurnal cortisol disruption in fibromyalgia syndrome. *Psychoneuroendocrinology*.

[82] Hoefgen B, Schulze TG, Ohlraun S (2005). The power of sample size and homogenous sampling: association between the 5-HTTLPR serotonin transporter polymorphism and major depressive disorder. *Biological Psychiatry*.

[83] Catley D, Kaell AT, Kirschbaum C, Stone AA (2000). A naturalistic evaluation of cortisol secretion in persons with fibromyalgia and rheumatoid arthritis. *Arthritis Care and Research*.

[84] Riva R, Mork PJ, Westgaard RH, Rø M, Lundberg U (2010). Fibromyalgia syndrome is associated with hypocortisolism. *International Journal of Behavioral Medicine*.

[85] Tanriverdi F, Karaca Z, Unluhizarci K, Kelestimur F (2007). The hypothalamo-pituitary-adrenal axis in chronic fatigue syndrome and fibromyalgia syndrome. *Stress*.

[86] Wingenfeld K, Heim C, Schmidt I, Wagner D, Meinlschmidt G, Hellhammer DH (2008). HPA axis reactivity and lymphocyte glucocorticoid sensitivity in fibromyalgia syndrome and chronic pelvic pain. *Psychosomatic Medicine*.

[87] Gold PW, Chrousos GP (2002). Organization of the stress system and its dysregulation in melancholic and atypical depression: high vs low CRH/NE states. *Molecular Psychiatry*.

[88] Selye H (1998). A syndrome produced by diverse nocuous agents. *Journal of Neuropsychiatry and Clinical Neurosciences*.

